# Stochastic Modeling of Mouse Motor Activity under Deep Brain Stimulation: The Extraction of Arousal Information

**DOI:** 10.1371/journal.pcbi.1003883

**Published:** 2015-02-26

**Authors:** Daniel M. Keenan, Amy W. Quinkert, Donald W. Pfaff

**Affiliations:** 1 Department of Statistics, University of Virginia, Charlottesville, Virginia, United States of America; 2 Laboratory of Neurobiology and Behavior, Rockefeller University, New York, New York, United States of America; 3 Laboratory of Neurobiology and Behavior, Rockefeller University, New York, New York, United States of America; 4 Laboratory of Neurobiology and Behavior, Rockefeller University, New York, New York, United States of America; University of Padova, Italy

## Abstract

In the present paper, we quantify, with a rigorous approach, the nature of motor activity in response to Deep Brain Stimulation (DBS), in the mouse. DBS is currently being used in the treatment of a broad range of diseases, but its underlying principles are still unclear. Because mouse movement involves rapidly repeated starting and stopping, one must statistically verify that the movement at a given stimulation time was not just coincidental, endogenously-driven movement. Moreover, the amount of activity changes significantly over the circadian rhythm, and hence the means, variances and autocorrelations are all time varying. A new methodology is presented. For example, to discern what is and what is not impacted by stimulation, velocity is classified (in a time-evolving manner) as being zero-, one- and two-dimensional movement. The most important conclusions of the paper are: (1) (DBS) stimulation is proven to be truly effective; (2) it is two-dimensional (2-D) movement that strongly differs between light and dark and responds to stimulation; and, (3) stimulation in the light initiates a manner of movement, 2-D movement, that is more commonly seen in the (non-stimulated) dark. Based upon these conclusions, it is conjectured that the above patterns of 2-D movement could be a straightforward, easy to calculate correlate of arousal. The above conclusions will aid in the systematic evaluation and understanding of how DBS in CNS arousal pathways leads to the activation of behavior.

## Author Summary

Brainstem and thalamic regulation of arousal has been studied experimentally since the mid 20-th century. Today, Deep Brain Stimulation (DBS) is used in the treatment of movement disorders, chronic pain, clinical depression, amongst others. At present, the proper choice of DBS parameters (frequency and strength of the electric stimulation), and how those parameters should be modified as conditions change, are not well understood. In this work, using motor activity as the observed response, a statistical framework is developed for such study, and a quantitative relationship between parameter values and response is established. Within this framework, a possible correlate of arousal, the rapid onset of spatial (two-dimensional) movement, is uncovered and also studied. One long-term hope for techniques such as DBS are that they could assist in the treatment of disorders of consciousness, by supplementing or replacing (e.g., in Traumatic Brain Injury) what should ordinarily be the appropriate endogenous stimulation.

## Introduction

Deep Brain Stimulation is currently being used in the treatment of Parkinson’s Disease, Disorders of Consciousness (DoC) and clinical depression [1–3]. The possibility that Deep Brain Stimulation (DBS) could be used to enhance brain arousal is a subject of immense interest, e.g., in traumatic brain injury (TBI). For example, in a human patient who had suffered DoC for more than seven years, DBS of the central thalamus was used successfully to aid in the recovery of his consciousness (Schiff et al (2007)) [4], Schiff (2010) [5]. From a fundamental neuroscientific point of view, this has been conceptualized as an elevation of generalized CNS arousal (Pfaff, 2006) [6]. In the mouse, locomotion is the most elementary of behavioral responses, and in this work we utilize such movement patterns as the basis of our inference relating DBS parameter changes to behavioral effects (Leshner and Pfaff (2011) [7], Benjamini et al (2011) [8], Quinkert et al (2010, 2011, 2012) [9–11]).

In the present study, the stimulations occur in the central thalamus (cf.Schiff et al [4]), over fixed 10 min intervals, every three hours, eight per day (four in light, four in dark). There are both stimulated and control (electrodes implanted but nonstimulated) mice. What is observed are the x- and y-coordinates of location, per second, over three days, with there being 12 hours of light, 12 hours of dark. The range of DBS parameters consist of three amperages and four frequencies, and were applied to each mouse over the three days (reported by Quinkert et al [9]). A common motor activity summary statistic is Total Activity, i.e., the total distance traveled over some fixed time interval (e.g., 10 min (600 sec)), or equivalently, Mean Activity (or Mean Speed): Total Activity divided by the number of time points (e.g., 600). Two questions that arise in the use of such statistics are, first, is there is a loss of important information in such Mean (or Total) Activity summarization; for instance, are important angular changes in direction or differences in the spatial range of movement, lost? Secondly, how does one calculate a standard error (or make a probabilistic assessment) for any statistic derived from such time-varying location data, doing so in a manner that can be justified? One needs to appropriately account for both local time-correlations, as well as broader circadian changes, otherwise the calculations may not be representative.

In a control mouse, or a stimulated mouse if there were no DBS effect, our basic assumption is that the time-varying processes constructed from the motor activity, are piecewise stationary. We will verify and apply piecewise stationarity for division into 3-hr segments, although longer periods could also be justified. We show that the autocorrelations within such a stationary segment die out after 20 minutes; segments of motor activity separated by 20 min can be assumed to be uncorrelated (or, in the present context, it is reasonable to assume independence). In the case of the stimulated mouse, we show that autocorrelations also die out after 20 min for regions sufficiently separated from a stimulation interval. The first stage of the analysis is to show that the stimulation is effective for at least one of the DBS parameter values. *In this analysis, all calculations are on segments separated by 20 min*; that is, our statistics for each animal are based on the ±80 minutes, centered at each stimulation time, leaving at least 20 min between any of the time intervals on which calculations are to be made. The statistic calculated on each (independent) segment results in a null hypothesis of no effect. A False Discovery Rate (FDR) thresholding then establishes that there is at least some DBS effect.

Once such a DBS effect is established, piecewise stationarity is used in a slightly different manner, since stationarity is being briefly perturbed by the stimulations to new steady-states, with possibly different stationary mean and/or the covariance structure for the 10 min stimulation intervals, than for nearby non-stimulated time intervals. That is, after establishing that there is some DBS effect, the determination of the relationship of DBS parameters to motor activity will need to be based on the collection of 10 min stimulation intervals, with the means and/or covariance structures for distinct such intervals possibly varying with the DBS parameter values. Differences in the stationarity structure for the different 10 min stimulation intervals, as we will see, result in significant differences in the variances for the calculated statistics, and hence does not allow for a traditional ANOVA formulation or nonparametric method. We will also consider differences in Mean Activity between light and dark, and piecewise stationarity will be the basis of that model.

As part of our analysis, we analyze speed and angular patterns and also decompose the movement into its randomly occurring epochs of 0-, 1-, and 2-dim movement. One-dimensional (linear) movement appears to be not unlike background noise in the present context, which is why the decomposition was formulated. The main ideas of the present paper important to neuroscience are: (**Hypothesis I**) The stimulation is effective; (**Hypothesis II**) It is 2-D movement, not 1-D movement, that occurs in response to stimulation, and there is a detectable relationship between DBS amplitude and frequency and the resulting movement; and, (**Hypothesis III**) It is 2-D movement, not 1-D movement, that differs between light and dark; and, stimulation in the light initiates a manner of movement (2-D movement) more commonly seen in the (non-stimulated) dark. To address these three hypotheses, we consider seven motor processes and their resulting statistics. For each, we calculate a forward 10 min mean (i.e., forward in time from each given sec) and a right minus left difference in 2 min means (at each sec); the later can detect rapid changes. The focus of several (3–4) of the statistics (those that are one-dimensional) is not on their power of information extraction, but rather the opposite; these statistics have basically no power and should be subtracted off, in order to obtain better statistics (those that are purely two-dimensional).

## Methods

### Ethics Statement

All animal procedures were in compliance with National Institutes of Health guidelines and approved by the Rockefeller University Institutional Animal Care and Use Committee.

### Experimental Setup

Details of methodology regarding neurosurgery and behavior have been published (Quinkert et al [9]). Briefly, mice were singly housed with food and water available and were subjected to a 12 h light/dark cycle. Stainless steel monopolar electrodes (0.3 mm diameter (Plastics One)) were insulated using polymide with 0.2 mm stripped from the electrode tips, with electrodes implanted bilaterally in the central thalamus. Stimulation was programmed and delivered by a four-channel stimulus generator (Multichannel systems STG2004). Stimulation epochs lasted for 10 min and occurred every 3 h over the course of 3 days. All stimulations were biphasic with a pulse width of 0.1 ms on both anodic and cathodic phases of the pulse. Three pulse amplitudes were applied, each for one day: 75, 100 and 125 *μ*A. Four pulse frequencies were selected from 50, 125, 175 and 225 Hz. The mice were euthanized and then a histological assessment of electrode placement was made, following data collection. Here, data are analyzed from novel points of view.

Nine mice were studied, with electrodes implanted in each; stimulation was applied in six mice (Mice 1–6), with three others used as controls (without stimulation, Controls 1–3). Four of the six stimulated mice (Mice 1–4) showed significant responses to the stimulations, whereas Mice 5–6 did not appear to respond at all; it was established via histological investigation that differences in electrode placement explained the nonresponsiveness of Mice 5–6. They were included in the initial analysis as a matter of completeness. The number of stimulations (*Nstim*) was 8 × 3 = 24 for mice 2, 4, 5 and 6, and was 23 for mice 1 and 3 (the recording of the 24-th stimulation period was not complete). Home cage activity data was collected by a 3D infrared monitor (Accuscan Instruments), which records the locations at the times of a detected change. The times between changes varied from the millisecond scale to that of multiple seconds; the data were interpolated to the one second scale, for computational purposes.

### Statistics That Do (Or Do Not) Extract Information from Mouse Movement

In the present mouse experiments, what is observed are the time-evolving locations (x- and y-coordinates) of the animal. The stimulation data is observed over 3 days, with N = 3*24*3600 = 259200 seconds. The stimulation times are denoted as {*S*_*k*_, *k* = 1, …, *Nstim*}, with Nstim being the number of stimulations. From the location data *r*(*t_i_* = (*x*(*t_i_*), *y*(*t_i_*)), *t_i_* = 1, …, *N*, one can calculate the (discrete-time) Velocity, Speed and Angle Direction processes:

$$\begin{array}{l} \underline{r}(t_{i})=(x(t_{i}),y(t_{i}))\\ \underline{V}(t_{i})=(V_{x}(t_{i}),V_{y}(t_{i}))=\underline{r}(t_{i+1})-\underline{r}(t_{i}),,t_{i}=0,\ldots ,N-1\\ M(t_{i})=\sqrt{x^{2}(t_{i})+y^{2}(t_{i})},\;{\underline{U}}^{x}=(1,0),\;\theta (t_{i})=\text{angle}(\underline{V}(t_{i}),{\underline{U}}^{x}) \end{array}$$ (1)

The angle *θ*(*t*_*i*_) is defined with respect to the positive x-axis (*U* ^*x*^) and is uniquely defined in [−*π*, *π*), with −*π* identified with *π* (i.e., the angles are on the unit circle). Since the location data are fully recoverable from the latter two and the initial location:

$$\underline{r}(t_{i})=\sum_{j=0}^{i-1}M(t_{j})\times (cos(\theta (t_{j})),sin(\theta (t_{j}))),$$ (2)

the Magnitude and the Direction of Angle processes are the basis for our analysis. Hence, we model the changing patterns of the following motor activity processes:

- *M*(*t*_*i*_): Speed, or Magnitude of the velocity, and by summing over any time interval one can obtain the Total Activity (i.e., Total Distance).
- *θ*(*t*_*i*_): Angle of direction as a function of time (relative to the fixed x-axis). Moreover, one can decompose these angles into two groups:
  - *θ* ^(*P*)^(*t*_*i*_): those that are Multiples of *π*/2 (e.g., continuation in same direction, a reversal or a perpendicular move), including movement parallel to the walls; and
  - *θ* ^(*NP*)^(*t*_*i*_): those that are Non-Multiples of *π*/2 (here, e.g., movement into the interior, non-parallel to a wall);
- Movement Pattern over a w = 30 sec window, calculated in a forward direction starting at each second, and decomposed into three groups (uniquely defined at each time point): (*D* ^(0)^(*t*_*i*_) = 0, for zero-dimensional movement)
  - *D* ^(1)^(*t*_*i*_): length of line segment containing the 1-dim movement (if points in time all lie on a line, and are not constant). One can determine if the movement over the 30 sec window beginning at *t*_*i*_, is one-dimensional, and if so, to calculate the length of its 1-D domain.
  - *D* ^(2)^(*t*_*i*_): area of the two-dimensional convex hull generated by the points (if they do not lie on a line). Because the two-dimensional path can, and often does, cross itself, defining the 2-D domain is not straightforward or necessarily well defined. A natural definition is the use of the convex hull. The convex hull of the points (*x*(*t*_*i*+*r*_), *y*(*t*_*i*+*r*_), *r* = 0, 1, …, *w* − 1), over a moving window of length w = 30 seconds, is determined and its two-dimensional area *D* ^(2)^(*t*_*i*_) is calculated.
  - *ID*(*t*_*i*_): identifies at each time whether the movement is zero-, one- or two-dimensional, by 0, 1 or 2 (based upon (i) and (ii), above).
- The Speed *M*(*t*_*i*_), when non-zero, can be decomposed into the Speed at times at which the movement is two-dimensional or one-dimensional: *M*(*t*_*i*_) = *M*_2*D*_(*t*_*i*_) + *M*_1*D*_(*t*_*i*_)
  - *M*_2*D*_(*t*_*i*_) = *M*(*t*_*i*_) if *ID*(*t*_*i*_) = 2, and 0 otherwise;
  - *M*_1*D*_(*t*_*i*_) = *M*(*t*_*i*_) if *ID*(*t*_*i*_) = 1, and 0 otherwise;
- One can define *A*(*t*_*i*_) to be the Total Activity over the 10 min window (*w*_1_ = 600) starting at *t*_*i*_ (i.e., the sum of *M*(*t*_*j*_) over the interval). For simplicity, we will use the Mean Activity $\bar{A}(t_{i})$ rather than Total Activity, in that the derivation of standard errors is more direct. The difference is merely one of scale. Based upon (d) above, summing over *M*_2*D*_(*t*_*i*_) and *M*_1*D*_(*t*_*i*_), respectively, $\bar{A}(t_{i})$ can be decomposed into the sum of 2-Dim and 1-Dim Mean Activity: (zero-dim movement adds zero)

$$\begin{array}{ll} \bar{A}(t_{i})=\frac{1}{w_{1}}\times \sum_{j=0}^{w_{1}-1}M(t_{i+j}) & =\frac{1}{w_{1}}\times \sum_{j=0}^{w_{1}-1}M_{2D}(t_{i+j})\;+\;\frac{1}{w_{1}}\times \sum_{j=0}^{w_{1}-1}M_{1D}(t_{i+j})\\ & =\;{\bar{A}}_{2D}(t_{i})\;+\;{\bar{A}}_{1D}(t_{i}) \end{array}$$ (3)

As we will see, it is the 2-Dim activity that differs most significantly between light and dark, as well as that which predominates in response to stimulation. That is, we will show that it is the statistics that extract 2-Dim information that are informative concerning responsiveness to DBS stimulation, and not those that are 1-dimensional.

**Fig 1:**
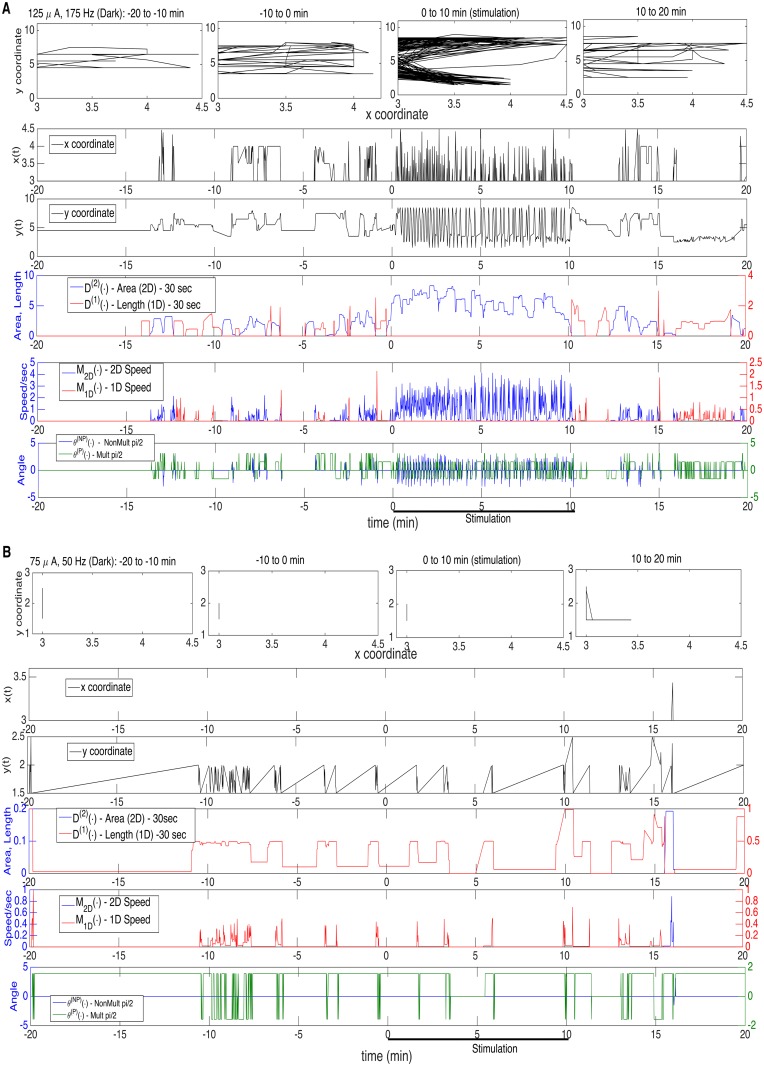
Motivation for the 1D and 2D Decompositions. Movement in Dark, for Stimulated Mouse 1, Before, During and After Stimulation. Two Panels: **A.** High DBS parameters: 125 *μ*A, 175 Hz.; **B.** Low DBS parameters: 75 *μ*A, 50 Hz. Four rows in each Panel. Row 1: actual time-varying two-dimensional position (per sec), over 40 min surrounding stimulation; Rows 2–3: individual x- and y-coordinate patterns over the 40 min; Row 4–6: the time-varying patterns of six (of the seven) motor processes; Row 4: 2D Area (Blue), 1D Length (Red); Row 5: 2D Speed (Blue), 1D Speed (Red); Row 6: Non-Multiple pi/2 angle (Blue), Multiple pi/2 angle (Green).

**Fig 2:**
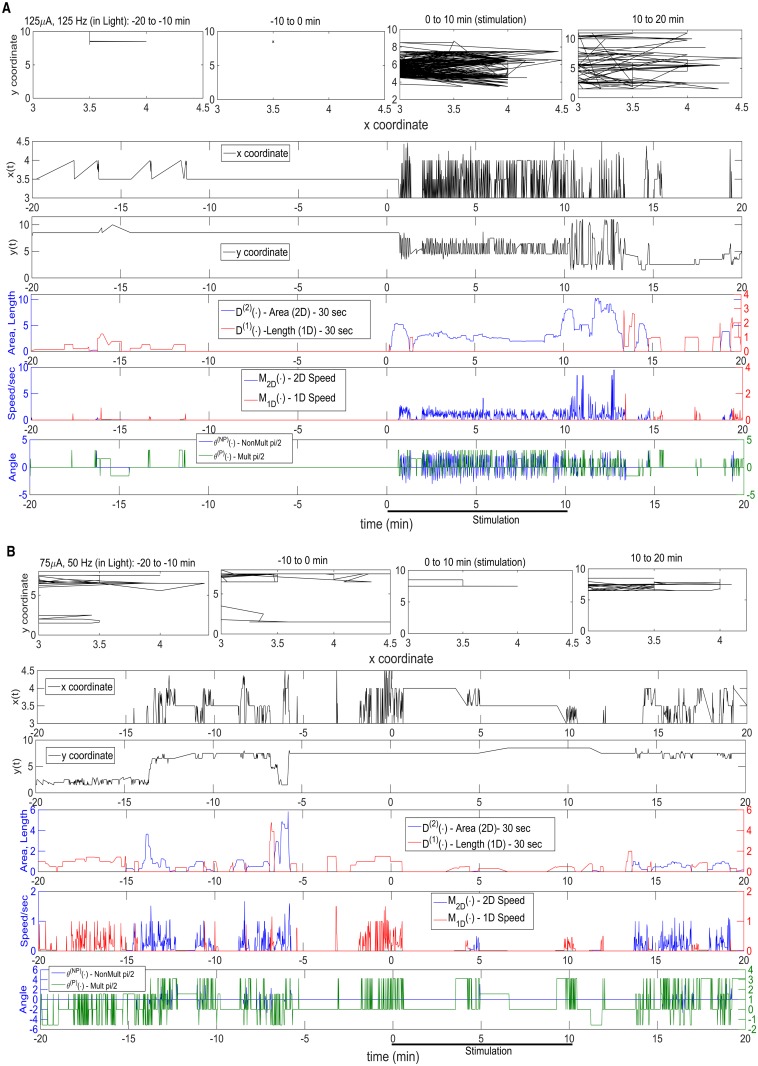
Same as Fig. 1, but with Movement in Light, for Stimulated Mouse 2. Two Panels: **A.** High DBS parameters: 125 *μ*A, 125 Hz.; **B.** Low DBS parameters: 75 *μ*A, 50 Hz. Four rows in each Panel. Row 1: actual time-varying two-dimensional position (per sec), over 40 min surrounding stimulation; Rows 2–3: individual x- and y-coordinate patterns over the 40 min; Rows 4–6: the time-varying patterns of six (of the seven) motor processes. Row 4: 2D Area (Blue), 1D Length (Red); Row 5: 2D Speed (Blue), 1D Speed (Red); Row 6: Non-Multiple pi/2 angle (Blue), Multiple pi/2 angle (Green).

In Figs. 1–2, there are two panels (**A** and **B**) in each, one for high (**A**) and low (**B**) parameter values; Fig. 1 is during the dark, Fig. 2 is during light, and they are for Stimulated Mice 1–2, respectively. In the first row of each panel are displayed the actual time-varying two-dimensional (x- and y-) position (per sec), over a sequence of four 10 minute intervals, starting 20 min prior to a stimulation, and including the 10 min stimulation interval and the 10 min interval following it. In the second and third rows are the individual x- and y-coordinate patterns, from which one can infer movement parallel or non-parallel to a wall. In the remaining rows are the above-described time-evolving motor processes, appropriately plotted; in row 4 are the time changing patterns of *D* ^(1)^(∙) (length, red) and *D* ^(2)^(∙) (area, blue); in row 5 are the analogous plots for Speed: *M*_1*D*_(∙) (1D, red) and *M*_2*D*_(∙) (2D, blue); and, in row 6, are the time-evolving *θ* ^(*P*)^(∙) (multiple of pi/2, green) and *θ* ^(*NP*)^(∙) (non-multiple of pi/2, blue). The seventh motor process, Mean Activity *M*(∙), being the sum of *M*_1*D*_(∙) and *M*_2*D*_(∙), was not plotted, for simplicity. The figures very much depict the motivation for the 1D and 2D decompositions, and their use in quantifying the stimulation response.

We will utilize two basic statistical calculations, defined below in expression (4): (1) a forward mean over a moving window of time (forward meaning that the value at time *t*_*i*_ is for the window starting at *t*_*i*_; the window width being *w*_1_ = 600 sec); and (2) a difference in means (i.e., that to the right minus that to left), for a moving window of time (window width being *w*_2_ = 120 sec, on each side). The latter statistic acts as a high-pass filter, detecting the rapid onset of movement. We denote the two statistics, respectively, as $\bar{X}(∙)$ and ${\bar{X}}_{RL}(∙)$:

$$\begin{array}{ccc} \bar{X}\left(t_{i}\right) & = & \frac{1}{w_{1}}\times \sum_{j=0}^{w_{1}-1}X\left(t_{i+j}\right)\;\;\text{and}\;\;{\bar{X}}_{RL}\left(t_{i}\right)=\frac{1}{w_{2}}\times \left(\sum_{j=0}^{w_{2}-1}X\left(t_{i+j}\right)-\sum_{j=-w_{2}}^{-1}X\left(t_{i+j}\right)\right) \end{array}$$ (4)

where *X* generically represents any of the following seven motor activity processes:

$$\begin{array}{c} A_{1D}\left(t_{i}\right),D^{\left(1\right)}\left(t_{i}\right),\theta^{\left(P\right)}\left(t_{i}\right),\theta^{\left(NP\right)}\left(t_{i}\right),A\left(t_{i}\right),A_{2D}\left(t_{i}\right),D^{\left(2\right)}\left(t_{i}\right) \end{array}$$ (5)

The first three are 1-Dim statistics *(1-D Activity, 1-Dim Length, Multiple of pi/2 angle)*. The next two are 2-dimensional *Non-Multiple of pi/2 angle, Total (1-D and 2-D, combined) Activity*, but are incomplete in certain ways (Results). The final two 2-dimensional statistics *(2-D Activity, 2-Dim Area)* are those that are of greatest potential interest.

**Fig 3:**
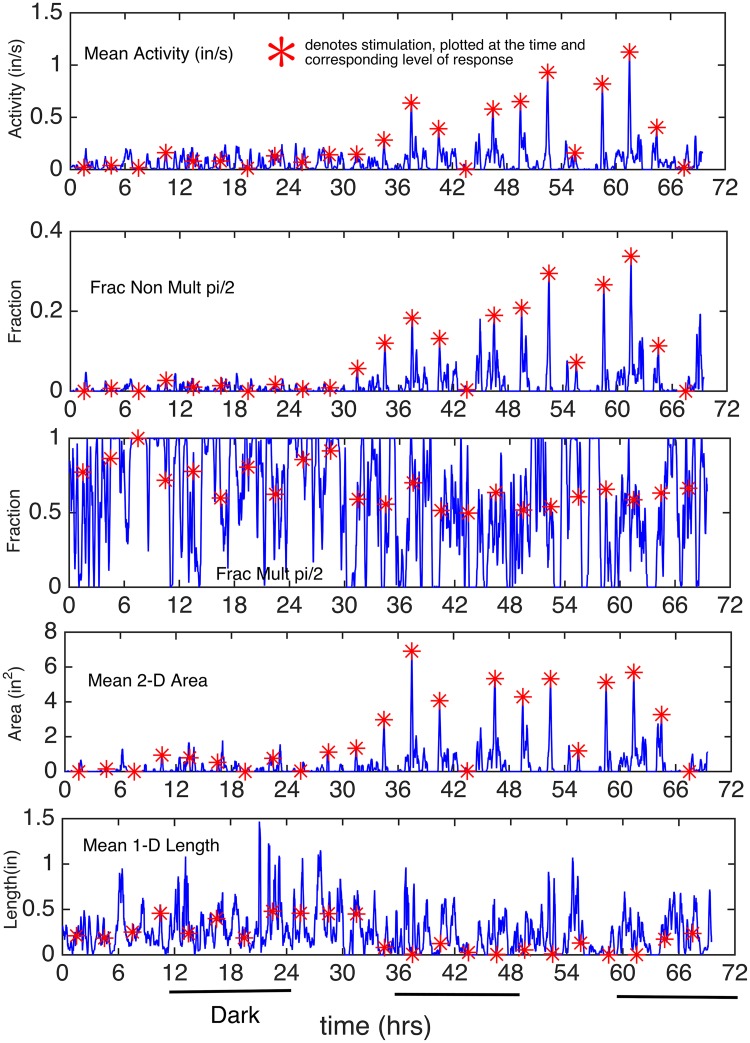
Plots of the Five Motor Activity Processes (for Mouse 1): Mean (or Fraction) Over 10 Min Interval, Starting at each Second. The five motor processes (one row for each) are: Mean Activity, Fractions of angles that are Non-Multiples of pi/2 and are Multiples of pi/2, Mean 2D Area and Mean 1D Length. The red asterisks denote the stimulation times, and are plotted at the height of the recorded response at the stimulation time to indicate the magnitude (or lack of) in response to the stimulus.

In Fig. 3, for Stimulated Mouse 1, the time-varying (per sec) statistics were averaged over a moving 10 min window starting at each second, moving second by second; the statistics were: mean activity, fraction of non pi/2 and fraction of pi/2 angles, mean area (of 2-D movement) and the mean length (of 1-D movement). The red asterisks denote the stimulation times, and are *plotted at the height of the recorded response at the stimulation time to accentuate the magnitude (or lack of) in response to the stimulus*. For the angle processes, the statistics calculated are fractions, rather than means.

### Piecewise Stationarity and the Local Autocorrelation Structure

Let *X*(*t*_*i*_) generically represent any of the following seven Motor Activity Processes given in expression (5), above. Our basic assumption is that, in the non-stimulated animal, or in the stimulated if there were no DBS effect (Hypothesis I), there is piecewise stationarity (in time) for motor processes under consideration, calculated from the location data *r*(*t_i_* = (*x*(*t_i_*), *y*(*t_i_*)), *t_i_* = 1, …, *N*. Below, we show such piecewise stationarity for the speed (*M*(*t*_*i*_)) process, but others (e.g., the angle process) can similarly be shown. Specifically, we assume that time can be decomposed into a finite set of time segments, for which the process of interest is assumed to be representable as a strictly stationary process on each segment. On different time segments, the structural parameters of stationarity (mean, variance, autocovariances, spectral density) are allowed to differ. We justify below the use of a decomposition into 3 hr time segments (although longer segments could be justified). Once it is shown that there is (some) DBS effect, piecewise stationarity will no longer hold in the same form, in that the local stationarity at the stimulation intervals is being perturbed by the stimulations to new brief steady-states on these 10 min stimulation intervals, or for possibly longer (as it returns to its original steady-state). In testing Hypothesis II, only the 10 min stimulation intervals will be used (they are now stationary at the perturbed stead-state, potentially different for different stimulation levels); in testing Hypothesis III, the stimulation intervals will be excluded. In modeling piecewise stationarity, as in stationarity, one can model from either the time- or frequency-domain. In the present work, we have chosen to use a time-domain approach, primarily because it made the analysis for the three hypotheses, as a whole, more unified. There has been a great deal of work on modeling time-varying spectra (see Ombao et al (2001) [12], Huang et al (2004) [13]).

**Fig 4:**
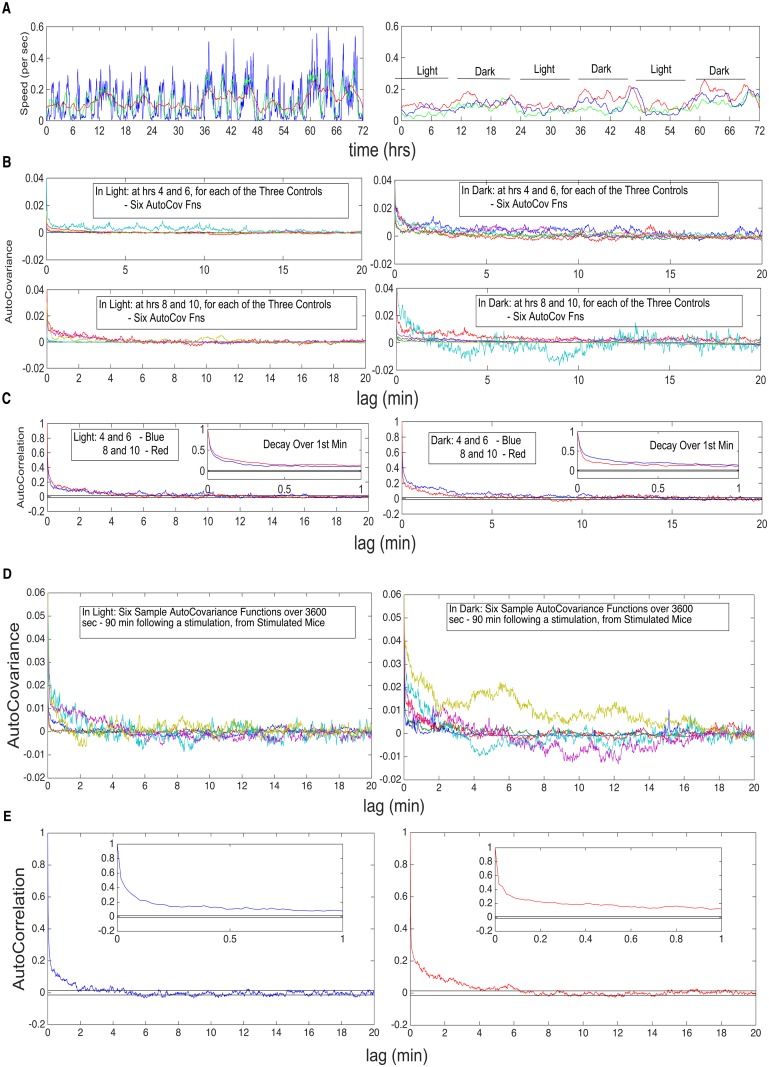
Verification of Loss of Correlation at the 20 Min Lag, for both Controls and Stimulated Mice. **A.** Top left, single control mouse, the local mean for speed process, over a moving window of 10 min (blue), 1 hr (green) and 3hr (red); top right, are the mean functions for all three controls, over a moving window of 3 hrs. **B.** The second and third rows display sample autocovariance functions (over 1 hr) for all three controls, starting at different hrs, in light and dark (see text for details). **C.** The fourth row displays the two autocorrelation functions calculated from the mean of the, respective, early (blue) and late (red) autocovariance functions. Loss of correlation identified at 20 min lag. Insets throughout the figure, show behavior of the autocovariance functions over the first minute of lag. **D.** Similar plots, but for the Stimulated Mice 1–4, at times in between the broad range of stimulation intervals, that are separated by 3 hrs.

We first establish the overall pattern of changing stationarity. In Fig. 4, there are four panels (**A–D**). In **A**, left, for a single control mouse, the local mean is calculated for the speed (*M*(*t*_*i*_)) process, over a moving window of width 10 min (blue), 1 hr (green) and 3hr (red); on the right, are the time-varying means for all three controls, over a moving window of width 3 hrs. Mouse movement typically consists in random bursts of movement, of random lengths, interspersed with low or no movement periods, of random lengths. One can view such patterns as being doubly stochastic, with the first level describing whether there is or is not movement. The mean patterns reflect such behavior, where the values locally can be quite variable. In **B**, there are two rows. The first displays the sample autocovariance functions (over 1 hr = 3600 sec)) for all three controls, starting at the 4th hr and at the 6th hr, in light (left) and dark (right), six functions in total. The second displays the same, calculated at the 8th and 10th hrs. The 4 and 6 hrs represent early behavior in the 12 hrs, whereas the 8 and 10 hrs represent late behavior for the 12 hrs. Panel **C** displays the two autocorrelation functions calculated from the mean of the, respective, early (blue) and late (red) autocovariance functions. The horizontal bands ($\pm 1.96/\sqrt{3600\times 6}$), which at a single lag is a confidence band about zero, are a standard time series practice to assess a “loss of correlation in time.” The result of Panels **A–C** of Fig. 4 is that, for the control mouse, the means are relatively stable during light and during dark, differing for the two; the same is true for the autocovariance structure, being relatively stable during light and during dark, but differing for the two. The autocorrelation patterns indicate that it is reasonable to assume that the statistics calculated on intervals that are *separated by 20 min* or more can be assumed to be uncorrelated (or, as we will, as independent). In the bottom panel (**D**) of Fig. 4, the autocovariances and autocorrelations are examined, for the stimulated mice 1–4, at times in between the broad range of stimulation intervals that are separated by 3 hrs. The autocovariances were calculated for 1 hr, starting 80 min after a stimulation interval. Again, one sees the same decay to negligible levels after 20 min, and hence, even in the stimulated case, intervals sufficiently separated can be assumed to be uncorrelated (again, in the present case, we will assume independence). Specifically, in **Hypothesis II**, where statistics are only calculated on the 10 min stimulation intervals, separated by 3 hrs, it is reasonable to assume uncorrelation (or, again, independence). Moreover, in Hypothesis III, in comparing 1D and 2D Mean Activity between light and dark, we will make calculations on three 3hr segments, separated by 1 hr, within light and within dark each, excluding the stimulation intervals and an additional 20 min following the stimulation. Hypothesis III concerns whether the means of the two (light, dark) are also different, in addition to their autocovariances.

**Fig 5:**
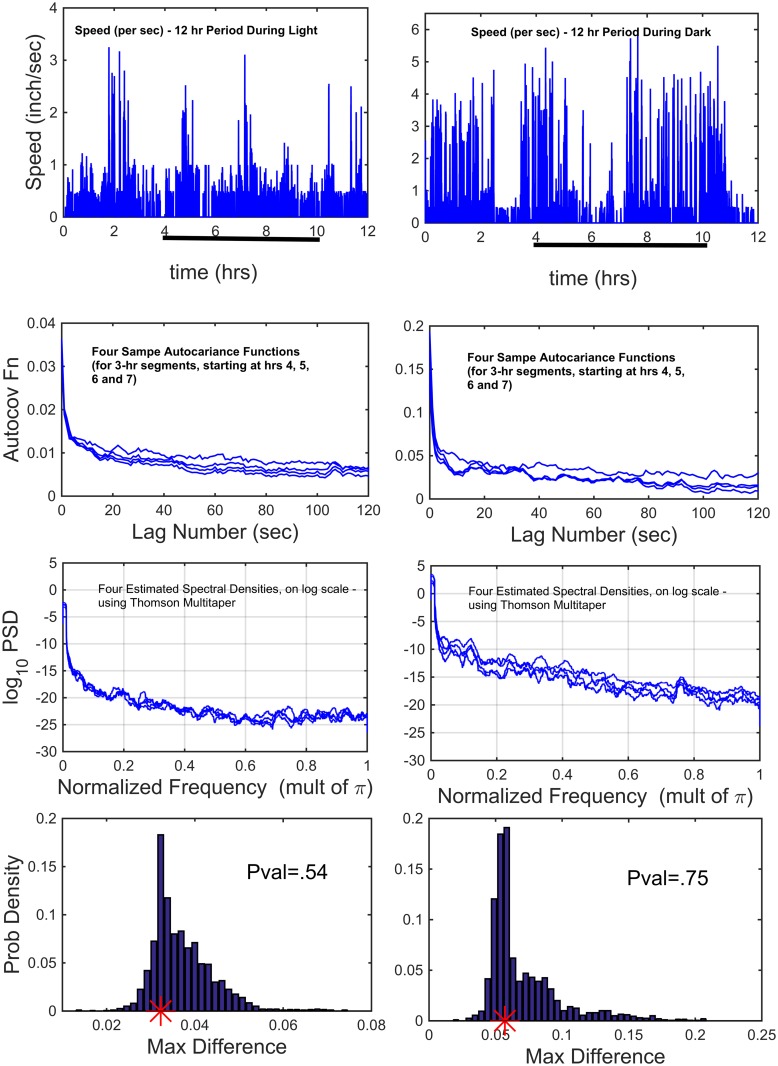
Test of Piecewise Stationarity: Comparing 3-hr Segments, Starting at Hrs 4, 5, 6 and 7, in Light and in Dark. Top Row: Speed (per sec) for a Control mouse over consecutive 12-hr light, 12-hr dark. Row 2: Sample autocovariance functions of the top row data (per sec), calculated over 3 hr periods, starting at hrs 4, 5, 6 and 7. Row 3: The estimated log spectral densities (using Thomson’s multitapering), for the four 3-hr time intervals. Row 4: The Diggle-Fisher test was applied (described in text), resulting in the probability histograms; the P-values for non-stationarity, in light and dark, are .54 and .75 (observed values for the data are the red asterisks).

In Fig. 5, top row are speed (per sec) data for a Control mouse (hence, non-stimulated). The left column displays 12-hrs in light and the right column 12-hrs in dark. In the second row are sample autocovariance functions of the top row data (per sec), calculated over 3-hr periods, starting at hrs 4, 5, 6 and 7. The most common approach to testing stationarity, has been spectral (squared modulus of the Fourier Transform), using the cumulative periodograms. The third row displays the estimated log spectral densities (using Thomson’s multitapering in the construction), for the four 3-hr time intervals. From the auto covariance functions, a dark versus light comparison, shows variances that differ (by a factor of more than five). However, within light and within dark, individually, the four variances show virtually no difference. Because of this, within light and within dark, we normalize the cumulative periodograms to a maximum of one (i.e., dividing by the variance). To test the equivalence of the four spectra, the Diggle-Fisher test (1991) [14] was performed, which is as follows. If the different sample spectra were all estimating the same true spectra, then shuffling the four spectral values at a given frequency, should statistically produce equivalent estimates. This is the basis of the test. One calculates the maximum difference at each frequency in the cumulative periodograms, and then the maximum of those over all frequencies. This is done for the actual observed cumulative periodograms and compared to the results for all the shuffling. The resulting histogram of the maximum difference (over all frequencies) of the shuffled cumulative periodograms, over 1000 shuffles, are displayed in the fourth row. The red asterisks denote the observed spectral maximum differences. The P-values for testing the hypothesis of stationarity during light is .54 and during dark is .75. Various other tests of stationarity have been proposed (e.g., Priestley and Subba Rao (1969)) [15], often as analogues of a Kolmogorov-Smirnov like test, which have proven difficult to use in practice.

### Time Series Basis for Testing Hypothesis I for the Motor Activity Processes

Our basic assumption is that, over a 3-hr period, there exists a strictly stationary process that describes the chosen motor process (e.g., the speed or angle processes), and that it satisfies a mixing condition, specifically *ϕ*-mixing (see Billingsley (1968)) [16], i.e., that there exists a function *ϕ*(∙), such that $\underset{n\to +\infty }{\lim}\phi (n)=0$, and for any two events, *F*_1_ and *F*_2_, *F*_2_ dependent upon the information up to time *m*, *F*_1_ dependent upon the information up to time *m*+*n*, *n* ≥ 0 and *P*(*F*_2_) > 0, they satisfy ∣*P*(*F*_1_∣*F*_2_)−*P*(*F*_1_)∣ ≤ *ϕ*(*n*). We also assume that its autocovariances are absolutely summable (and hence a continuous spectral density exits). Phi(*ϕ*)-mixing is a very weak assumption which describes the rate at which time-dependency dies out. From Figs. 4–5, this is a very reasonable assumption of the dying out of the dependencies. Since $\bar{X}(∙)$ and ${\bar{X}}_{RL}(∙)$ are finite linear filters of *X*(∙), they also are strictly stationary and satisfy the same (form of) mixing condition. Let $F_{\bar{X}}(t_{j})$ denote the marginal distribution of $\bar{X}(∙)$ at a (arbitrary) single time point *t*_*j*_, which by strict stationarity is the same at all times *t*_*j*_ (in the segment of stationarity). Under the assumption of *ϕ*-mixing, the empirical distribution function *F*_*n*_(∙): $\begin{array}{ccc} F_{n}\left(x\right) & = & \frac{1}{n}\sum_{j=S_{k}-80*60}^{S_{k}+80*60}I_{\left[\bar{X}\left(t_{j}\right)\leq x\right]}\;\;,\;\text{where}\;n\;\text{is}\;\text{the}\;\text{number}\;\text{of}\;\text{terms}\;\text{in}\;\text{the}\;\text{sum}, \end{array}$ is asymptotically equivalent to $F_{\bar{X}}(∙)$ (see Billingsley (1968)) [16]. Specifically, there is uniform weak convergence, with $sup_{x}\sqrt{n}(\;F_{n}(x)-F_{\bar{X}}(x)\;)$ converging to a Gaussian process (indexed by *x*). Hence, we have that $\underset{n\to +\infty }{\lim}F_{n}(x)=F_{\bar{X}}(x)$, uniformly in *x*. As a consequence, probability calculations under *F*_*n*_ are asymptotically equivalent to those under $F_{\bar{X}}$.

**Fig 6:**
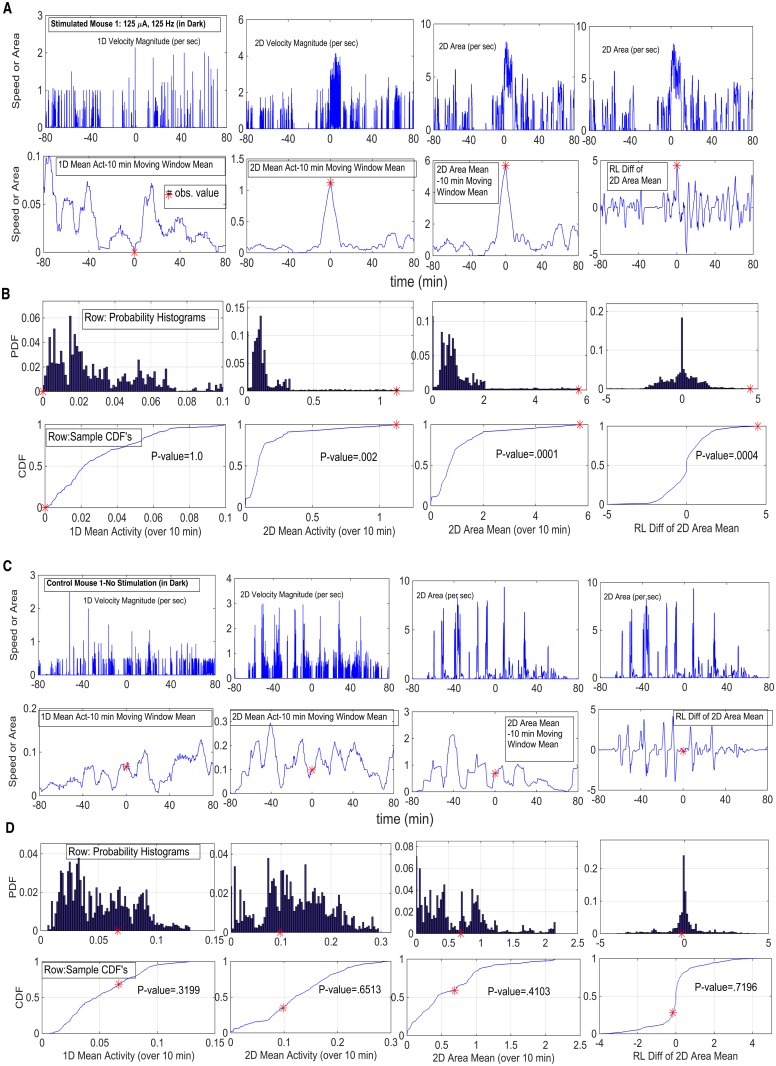
Representative P-value Calculations for Four Statistics in Hypothesis I: 1D and 2D Velocity Magnitudes, 2D Area and R-L Diff of 2D Area. **A.** Stimulated Mouse, in Dark; DBS parameters 125 *μ*A, 125 Hz. Row 1: 1D and 2D Velocity and 2D Area Magnitudes; Row 2: Values for each statistic, based upon the translations (restricted shuffling procedure); observed value denoted by red asterisk. **B.** Probability histograms and cumulative distribution functions; observed value denoted as red asterisk and P-value indicated (see text for more detail). **C–D.** Control Mouse, No Stimulation; in Dark. Row Descriptions same as in Panels A–B.

Since *S*_*k*_ is a fixed time (the k-th stimulation time onset), if there were no effect due to the stimulation at time *S*_*k*_, then $\bar{X}(S_{k})$ is random with the probability distribution $F_{\bar{X}}$. Evidence against the hypothesis that there is no effect due to the stimulation at time *S*_*k*_, can hence be measured by the probability of observing a value greater than or equal to $\bar{X}(S_{k})$ under *F*_*n*_, i.e., a P-value for each *k*, *k* = 1, …, Nstim. This comparison can be interpreted as a permutation test except that the permutations are restricted to being translations (or rotations if viewed on a circle). This restriction is a direct consequence of the test adhering, as is necessary, to the piecewise stationarity. The same results apply to the empirical distribution function for ${\bar{X}}_{RL}$. Thus, for **Hypothesis I**, the question is (where $\bar{X}$ represents any of the seven motor processes): Is $\bar{X}(S_{k})$, for a fixed *k*, *k* = 1, …, Nstim, significantly greater than most $\bar{X}(t_{j})$, for *t*_*j*_ in the ±80 min (160 min) period centered at *S*_*k*_? If so, this is (probabilistic) evidence that the stimulation caused a change in motor activity. The same question applies to ${\bar{X}}_{RL}(S_{k})$. In each case, one calculates the proportion of the values that lie above $\bar{X}(S_{k})$ and ${\bar{X}}_{RL}(S_{k})$, respectively. Representative P-values (k = 1, …, NStim) are presented in Fig. 6 for Mouse 1 and Control 1. If one wishes to make an assessment of the effect due to a given amperage level (i.e., the level over a given day), one can apply a False Discovery Rate (FDR) procedure (presented in **Results**) (Benjamini and Hochberg (1995) [17], Benjamini and Yekutieli (2001) [18]).

### Standard Error for $\bar{A}(S_{k})$, the Mean Activity (and its 2-D and 1-D components), over a Stimulus Interval

In the case of **Hypothesis II**, once **Hypothesis I** has been affirmed (that there are motor effects due to the stimulations), then one must establish the standard error for the statistics, using only that particular 10 min stimulation interval, since the structure on that interval can now be quite different from that of neighboring non-stimulation intervals or other 10 min stimulation intervals (corresponding to different DBS parameter values). Hence for the Mean Activity (and its 2D and 1D components), obtained by summing over the 10 min stimulation interval, one needs to base its standard error calculation on the stationarity of the speed over same 10 min stimulation interval. For a stationary time series with a covariance function that dies out sufficiently fast so that the spectral density exists and is continuous (which occurs under our assumptions), the asymptotic variance of a sample mean (sample size n) of the process is:

$$\underset{m\to +\infty }{\lim}(\gamma (0)+2\sum_{h=1}^{m-1}\left(1-\frac{h}{m}\right)\gamma (h))$$ (6)

and the standard approximation to this, is to replace the covariances with their sample estimates, using a number of terms (*m*) of an order less than *n*. A standard practice is to use as the number of terms (*m*), the square-root of *n* (see Shumway and Stoffer (2011) [19]). Hence, the approximation can be applied to the various motor processes (expression (5)), e.g., $\bar{A}(S_{k})$, ${\bar{A}}_{2D}(S_{k})$ and ${\bar{A}}_{1D}(S_{k})$, ${\bar{A}}_{1D}(S_{k}),\;$ *k* = 1, 2, …, *Nstim*. One might ask if it is more accurate to fit a time series ARMA(p, q) model to each stimulation 10 min interval, estimate the ARMA parameters and to then use covariance estimates based upon these finite number of parameters. For finding standard errors of most time series parameter estimates, that approach is superior; but for the sample mean, because of its linear construction, there is no improvement, in that the use of the sample covariances in the above construction, produces an asymptotically efficient estimator (Grenander and Rosenblatt (1957) [20], Priestley (1981) [21]). In testing Hypothesis II, the above standard errors for the motor processes are utilized (Results, Fig. 8).

### Differences Due to Light and Dark in terms of 1D and 2D Mean Activity Responses to Stimulation

In order to identify the effect of dark versus light on one- and two-dimensional movement patterns, we break, for each of the 3 days, the 12 hrs (720 min) of light and dark each into three 3hr segments, with 1 hr between: 30 to 210, 270 to 450, 510 to 690. We do this for each of the nine mice. This breaks the 3 days into a sequence of eighteen 3hr segments, each separated by 1hr. In addition, certain times are excluded: those times during a stimulation and the 20 min following a stimulation (for the Stimulated Mice), and the ±30 min at a light/dark transition are excluded by construction (they are within the 1 hr separating the segments). On each segment we calculate the 1D and 2D Mean Activity (${\bar{A}}_{1D}$, ${\bar{A}}_{2D}$). We assume piecewise stationarity for the 3 hr segments. The sample autocovariances and means are calculated on each 3 hr segment. We do this for the 1D Mean Activity and the 2D mean Activity, separately. The variances of the means for each 3hr segment are calculated, as they were for the 10 min stimulation intervals, by applying expression (6) above. We then average these 3hr-based means over the dark and over the light, and take the difference. What we wish to test is whether or not there are differences in the means in light versus dark, for 1D and for 2 D Mean Activity. Let *Y*_1*Dim*, *D*−*L*_ and *Y*_2*Dim*, *D*−*L*_ denote these differences in means. Thus, we obtain values *Y*_1*Dim*, *D*−*L*_, EstVar(*Y*_1*Dim*, *D*−*L*_), *Y*_2*Dim*, *D*−*L*_ and EstVar(*Y*_1*Dim*, *D*−*L*_). The test statistics are:

$$Y_{1Dim,D-L}/\sqrt{\text{EstVar}(Y_{1Dim,D-L})}\;\text{and}\;Y_{2Dim,D-L}/\sqrt{\text{EstVar}(Y_{2Dim,D-L})}$$ (7)

For each of the nine mice, a P-value can be calculated for the dark/light comparison, for 1D and 2D Mean Activity, and FDR analysis of the P-values is applied. These values are given in Fig. 9A–B, as part of the testing of Hypothesis III.

## Results

The important **ideas** of the present paper are: (**Hypothesis I**) The stimulation is effective. The methodology confirms that the resulting movement at the stimulation times (under appropriate DBS parameters) is in fact stimulation-driven, delineating it from merely being coincidental endogenously-driven movement; (**Hypothesis II**) It is 2-D movement, not 1-D movement, that occurs in response to stimulation, and, the effects due to the three amperages (75, 100, 125 *μ*A) are statistically increasing in value and distinguishable. Moreover, there is a significant synergism at the combination of 125 *μ*A and 125 Hz. In terms of the four frequencies, the effect due to 50 Hz was less than that for each of 125, 175, 225 Hz. There is not a significant response in 1-D movement to the stimulation; and, (**Hypothesis III**) It is 2-D movement, not 1-D movement, that differs between light and dark, and finally, stimulation in the light initiates a manner of movement (2-D movement) more commonly seen in the (non-stimulated) dark.

In order to establish the above ideas, we utilized two basic statistical calculations, defined above in **Methods**: (1) a forward mean over a moving window of width 10 min; and (2) a difference in right minus left means with moving window of width 2 min. The calculations are applied to each of seven motor activity processes:

$$A_{1D}\left(t_{i}\right),D^{\left(1\right)}\left(t_{i}\right),\theta^{\left(P\right)}\left(t_{i}\right),\theta^{\left(NP\right)}\left(t_{i}\right),A\left(t_{i}\right),A_{2D}\left(t_{i}\right),D^{\left(2\right)}\left(t_{i}\right).$$

The first three are 1-Dim statistics *(1-D Activity, 1-Dim Length, Multiple of pi/2 angle)*; we show that these statistics, are best removed from the resulting statistics, leaving only 2-dimensional components. The next two are 2-dimensional (*Non-Multiple of pi/2 angle, (Total (1-D and 2-D, combined) Activity*), but lack certain strengths, e.g., the Total Activity still has the 1-dim activity as a component, and the angle calculation has 2-dim information but has no velocity magnitude information. The final two 2-dimensional statistics *(2-D Activity, 2-Dim Area)* are those that have the greatest strength. (For the angle processes, the statistics calculated are fractions, rather than means.)

For **Hypothesis I**, to test that there is a response to (at least some of) the stimulations, the asymptotic distributions of our test statistics, under the assumption of piecewise stationarity, were derived in **Methods**. Statistically, one is going to calculate a single value, based upon that particularly given 10 min interval of stimulation. The starting point of each stimulation interval is surrounded by a ±80 min larger interval, with these larger intervals all separated from one another by at least 20 min. Hence, calculations on each (based upon results of Methods) are independent of one another. One wishes to show that the value of the statistic calculated for the 10 min stimulation interval is significantly different from the same calculation at an arbitrary point in the surrounding interval. The mouse is being stimulated by environmental cues all the time. One needs to show that the motor activity at the stimulation time was not just coincidental endogenously-drive movement, but rather significantly different from such. One cannot permute because of time dependency, and bootstrapping in a stationary context is difficult and involves various heuristic choices (Kunsch (1989) [22], Lahiri (2003)) [23]). However, one can imagine, for any value in the surrounding 160 min interval, that one makes the same calculation for a 10 min interval starting at any point in the 160 min. Calculations at the limits of the 160 min interval, are made in a wrap-around manner (i.e., the interval is viewed as a circle). Such wrapping around is a standard time series/Fourier procedure that has no asymptotic effect; so doing allows us to make the calculations and keep the different 160 min intervals sufficiently separated. The sample cdf of all such translations (rotations) is asymptotically derived in **Methods**, under the stated conditions of *ϕ*-mixing and absolute summability of autocovariances, which are very weak assumptions. That is, the appropriate test statistic is the restriction of permutations to just those that are translations (i.e., rotations, if viewed as a circle); only the translations adhere to the local time invariance of stationarity. In Fig. 6, representative calculations, including the final P-values of the test statistics, are displayed for one stimulation mouse (Panels **A–B**) and one control mouse (Panels **C–D**). There are four rows for each mouse. In the first row are shown the raw data on which each of four statistics are to be calculated; the second row shows the collection of values for each statistic, based upon the translations, plus the observe value of the statistic is displayed as a red asterisk. The third and fourth rows show, respectively, the resulting probability histogram and cumulative distribution function, with the observed value displayed as a red asterisk and the P-value is indicated.

**Fig 7:**
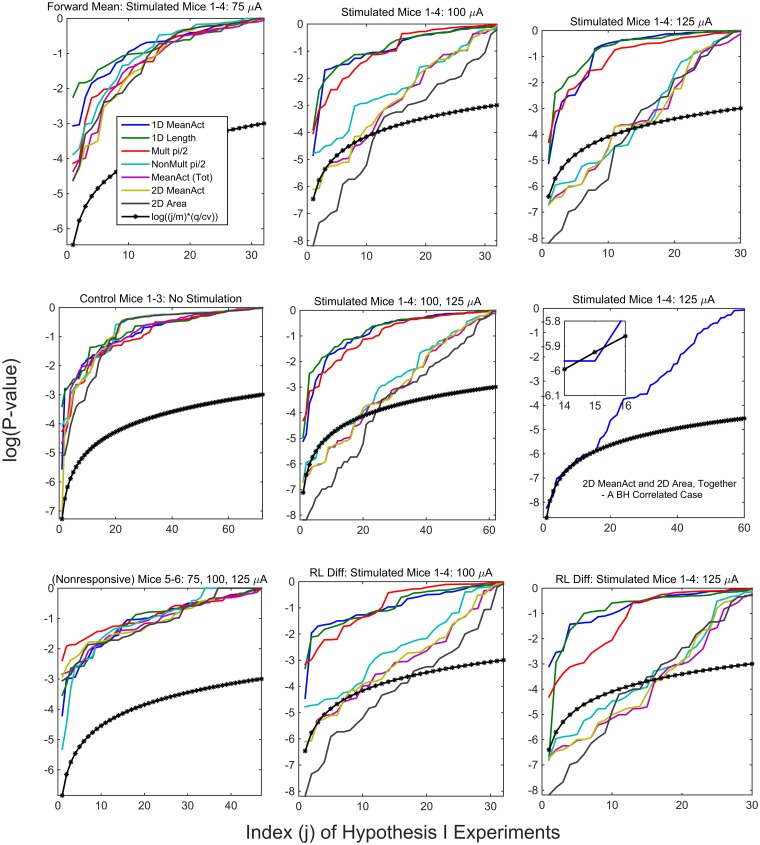
False Discovery Rate Analysis for Hypothesis I: Plots of the P-values for null hypotheses, for the Stimulated Mice 1–4, the control Mice 1–3 and the Nonresponsive Mice 5–6. Two conclusions: The three 1-dimensional statistics never go below the threshold function in any of the cases; in the combined 100, 125 *μ*A plot for the Stimulated Mice 1–4 (middle plot of Row 2), and the four 2-dimensional statistics each have between 20 and 25 significant tests out of the combined 62.

In Fig. 7, Hypothesis I is tested, and the P-values for all mice and motor process statistics are summarized; the results of Fig. 6 are contained within these. Because the calculations are all made on distinct intervals separated by 20 min or more, as described in **Methods**, the calculations can be assumed to be uncorrelated or, more specifically, independent. For a given mouse, there are 24 (or 23 for two) stimulation intervals; each stimulation interval produces a null hypothesis for no DBS effect at those particular parameters (Amperage, Hz, light/dark). For a chosen statistic, a P-value is obtained for each interval, for each mouse. In this multiple testing setting, we use a False Discovery Rate (FDR) thresholding at *q* = .05, to assess the evidence of significance. First, though, the first eight stimulation intervals are for amperage 75*μ*A. This value was included as a baseline, with little expectation of response, but rather to be used as a reference point for the primary two: 100, 125 *μ*A. If one is assessing the strength of evidence, it does not seem appropriate to include these eight in any overall assessment, but to be considered separately. [It is analagous to including experiments with a placebo to determine if there is some physiological response to an agonist.] For simplicity and compactness of display, we have shown, in some of the subplots, the results simultaneously for all seven of the motor processes. In Fig. 7, displayed are the sorted P-values (uncorrected) for *m* null hypotheses, and the thresholding function (*j*/*m*) * (*q*/*cv*); in the independence case, *cv* = 1, and in the correlated case, cv is the sum of reciprocal indices (Benjamini and Hochberg (1995) [17], Benjamini and Yekutieli (2001) [18]).

As stated above, our calculations are independent for distinct intervals. In Fig. 7, first row, we display the three amperages 75, 100 and 125 *μ*A, separately. One can argue that the three are different experimental conditions. In the second row, middle plot, the 100 and 125 *μ*A null hypotheses are combined. As has been stated previously, one aspect of the work is to show that the three 1-dimensional statistics do not reflect DBS responsiveness, and should in fact be removed within appropriate statistics. The two statistics: Non-multiple of pi/2 (contains 2D information, but lacks information about velocity magnitude) and the Mean Activity (combining 1D and 2D) both lacked strength. The remaining two statistics are those of primary interest: 2D Mean Activity (${\bar{A}}_{2D}(S_{k})$) and the Mean 2D Area (${\bar{D}}^{\left(2\right)}(S_{k})$). We are not trying to choose between the seven statistics; each identifies separate and distinct information. However, we include a plot (second row, rightmost) of the null hypotheses for the two primary statistics, which, since they are correlated, we use FDR in the correlated case. In the second row, leftmost plot, we consider the 3 controls. In the third row we display the results for the two nonresponsive mice (Mice 5–6), on the left. The other two subplots consider the Stimulated Mice 1–4, and the second form of the calculations: Difference between the Right and Left Means, at each point; this can be viewed as a high pass filter or as a change-point detector. An expression of the strength of evidence in a given subplot is the number of significant hypotheses. One statement of the strength of evidence for at least some detectable DBS effect, would be the combined 100, 125 *μ*A plot for the Stimulated Mice 1–4, which is the middle plot of row 2. There, the four 2-dimensional statistics each have between 20 and 25 significant tests out of the combined 62 (32+30). In all of the cases: Stimulated Mice 1–4, Controls 1–3, and Nonresponsive Mice 5–6, the three 1-dimensional statistics never go below the threshold function.

The results in Fig. 7, with respect to **Hypothesis I**, are that: (i) there is no measurable response, for any of the seven processes, to the stimulations at the lowest ampere level of 75 *μ*A; this was not unexpected, in that it was chosen to hopefully identify a baseline level; (ii) the (total) Mean Activity, Mean Activity 2D, the Fraction of Non-Multiples of pi/2 Directions and the Area 2D, represent measurable stimulation responses at the 100 and 125 *μ*A; and, (iii) the Mean Activity 1D, Fraction of Multiples of pi/2 Directions and the Length 1D, show virtually no response.

**Fig 8:**
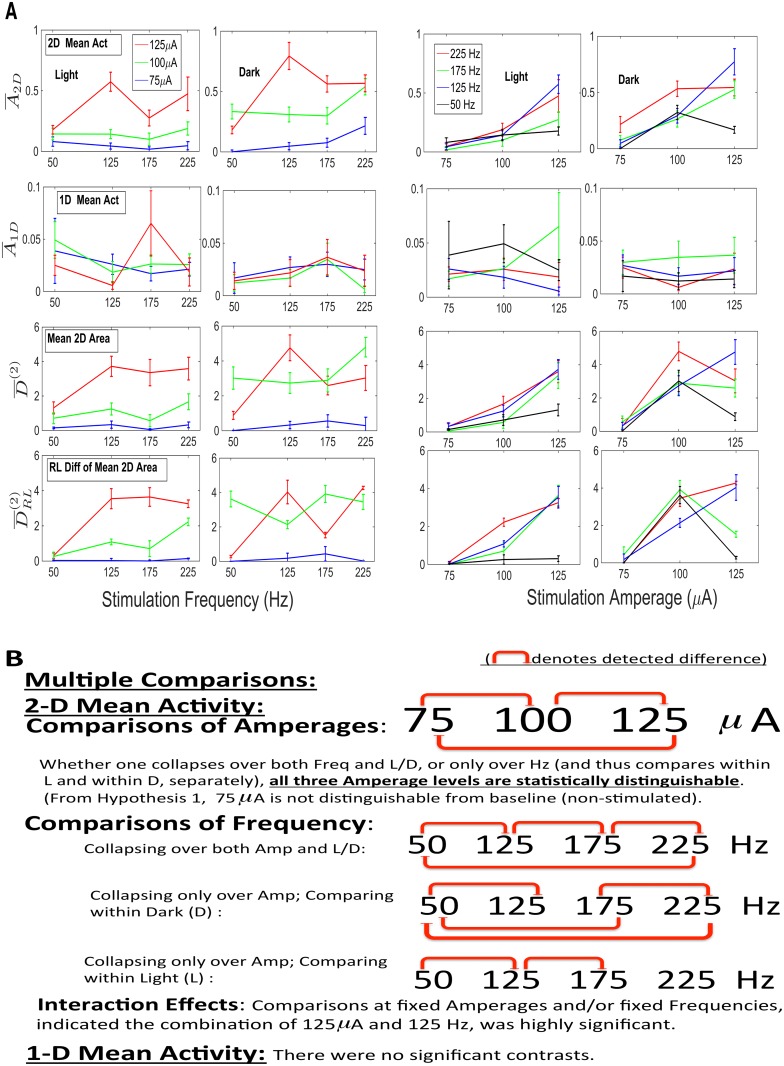
Hypothesis II. A. The magnitudes of four statistics (1D and 2D Mean Activity, Mean 2D Area and RL Diff in Mean 2D Area) are plotted as functions versus frequency (Columns 1–2), and functions versus amplitude (columns 3–4), in light and in dark. Error bars are (±) one SEM. **B.** Summary of the results for the various multiple comparisons, at the .05 level, in the case of two of the statistics (2D and 1D Mean Activity). All three amperages are statistically distinguishable, and there is a highly significant synergism between 125 *μ*A and 125 Hz.

Once one has shown that there are motor responses specifically due to stimulation, one then proceeds to test (**Hypothesis II**) that these responses can be related to the stimulation parameters. To calculate a standard error for the stimulated response, one is restricted to using only the data in the 10 min stimulation interval itself, in that the distribution of the test statistic is now known to be different outside the stimulation interval. A time series method was presented in the **Methods** to calculate the standard errors for the statistics over each of the 10 min stimulation intervals (intervals separated from another by 3 hrs). One can combine these means and (unequal) standard errors across animals, to obtain overall means and standard errors. The availability of these standard errors allows one to make multiple comparison calculations, in order to assess differences in the responses with respect to amplitude (*μ*A) versus frequency (Hz) and versus light or dark (L/D). Because the standard errors differ significantly across amplitude and frequency, traditional methods such as Analysis of Variance or the nonparametric Kruskal-Wallis test, both of which require a constant variance, cannot be applied. There is a certain degree of robustness, but the differences here are significantly beyond that (a factor 5–10, at times) (Scheffe (1959)) [24]. An alternative is still available. The statistical basis for our analysis is a multiple comparisons test, Dunnett’s C test, for which the variances are allowed to be unequal, as are the sample sizes (Dunnett (1980) [25]. In Fig. 8A, the data, consolidated over Mice 1–4, are displayed for four different statistics: 1D and 2D Mean Activities, Mean 2D Area and the RL Mean Difference of 2D Area. In Fig. 8B, a summary of the multiple comparisons (at *α* = .05) is presented for the the 2D and 1D Mean Activities. The results of the other two 2D statistics are similar to those of the 2D Mean Activity.

In summary, for Hypothesis II, the mean activities due to the three amperages were statistically distinguishable (and increasing with respect to amperage), whether one collapsed over L and D or compared within L or within D. From **Hypothesis I**, one knows that 75 *μ*A could not be delineated from non-stimulation, and now it is shown, statistically, that 100 *μ*A results in an increase in activity, and 125 *μ*A in an even greater increase. As for comparisons of frequencies, they differed as to whether L and D were combined or not. A general statement (at *α* = .05) is that in dark, 50 Hz was statistically distinguishable from all three higher values (125, 175 and 225 Hz). If one considers nonlinear interactions between amperage and frequency, there is a significant synergistic effect at the combination of 125 *μ*A and 125 Hz, an important, practical conclusion. Again, all of the above results were based upon multiple comparison procedures.

**Fig 9:**
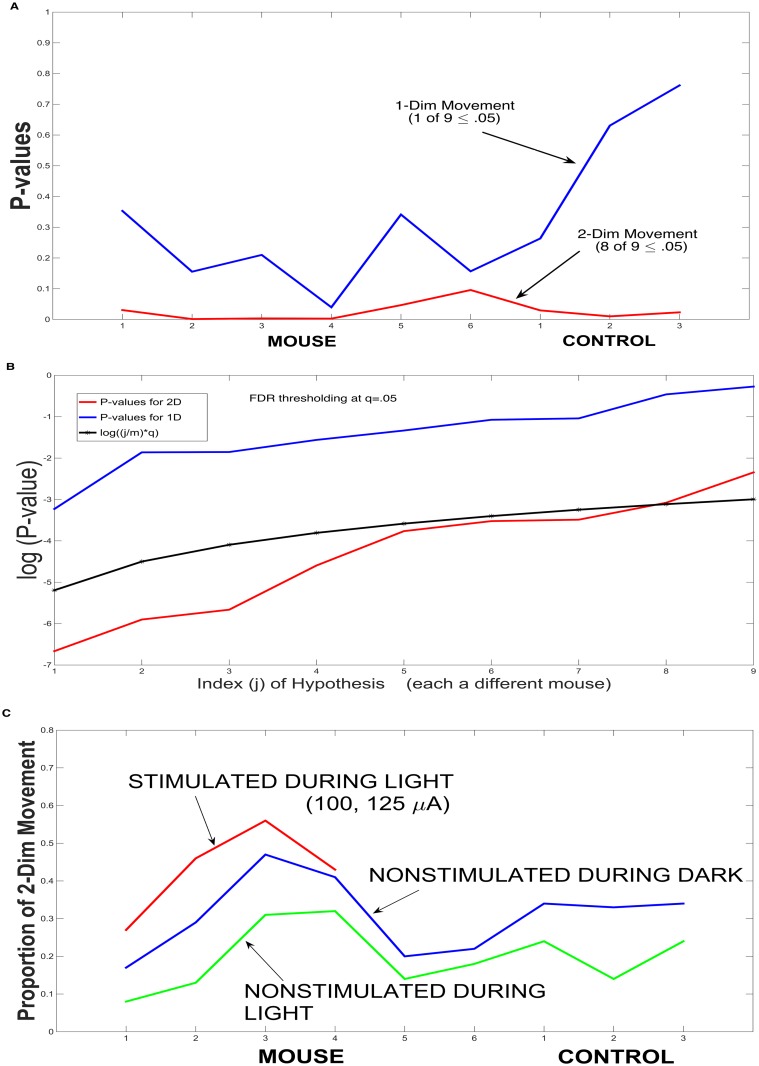
Hypothesis III. A Test of Dark versus Light Patterns for 2-D and 1-D Mean Activity. **A.** A plot of P-values, for each of the nine mice, for the difference between dark and light for 1-D movement and for 2-D movement. **B.** False Discovery Rate (FDR) thresholding at q = .05, for the null hypotheses in panel A. For 2-D, eight of the nine hypotheses were significant; for 1-D, none of the nine were significant. **C.** For each of the nine mice, plotted are the proportion of 2-D movement in light during non-stimulation (in green), in dark during non-stimulation (in blue), and, finally, the proportion in light during stimulation, for Mice 1–4. The plots provide additional evidence that stimulation in light initiates movement that is more naturally nocturnal.

**Hypothesis III** is that stimulation initiates a pattern of movement that is more common to dark. Specifically, we show that 2-D movement bursts are more natural in the dark and that stimulation of sufficient strength in the light initiates a 2-D movement burst of the form that occurs in the non-stimulated dark state. The analysis for this hypothesis is based upon a comparison of the data, for all nine mice. To compare the 2-Dim and 1-Dim Mean Activities during light and during dark, test statistics were constructed (in expression (7)) (comparisons of the means during dark with those during light) and their standard errors were calculated, based upon estimated autocovariance functions (see Methods). P-values were calculated based upon the test statistics. For two-dimensional movement, using nine mice (six stimulated (two, non-responsive) and 3 non-stimulated controls), eight of the nine were statistically significant at.05 for the Day-light comparisons. For one-dimensional movement, only one of the nine was significant (at.05) (Fig. 9A). The strength of the evidence in such a multiple testing setting (nine null hypotheses each for 1D and 2D) was evaluated by a FDR thresholding at *q* = .05, with eight of the nine hypotheses being significant for 2D, and none of the nine hypotheses were significant for 1D (Fig. 9B).

The evidence is very strong for the day/light difference in two-dimensional movement. For one-dimensional movement, it is a matter of interpretation; at most, it would suggest a very weak circadian effect. Finally, in Fig. 9C, evidence is given that stimulation during light initiates movement representative of non-stimulated nocturnal movement (which from Fig. 9A–B is 2-D movement). Plotted are the fractions of 2-D movement during non-stimulated light and non-stimulated dark (for Mice 1–6, Controls 1–3), and the fractions during stimulated light and stimulated dark (for Mice 1–4). The plots reveal that similar changes occur during stimulated light (Mice 1–4) and non-stimulated dark, as compared to non-stimulated light, providing additional evidence that stimulation in light initiates movement that is more naturally nocturnal.

We have shown that Deep Brain Stimulation does initiate motor activity in response to stimulation, distinguishing it from what otherwise might have been just the coincidental occurrence of the continuously occurring stop-and-start movement of the mouse. We established that there is an increasing level of response to an increasing level of amperage, and increased response to the three higher frequencies. Significant synergism at the 125*μ*A, 125 Hz combination was uncovered. The responses were shown to be those corresponding to 2-dim movement, not 1-dim movement. That 2-dim movement is much more common in dark than in light, was quantified. An important identification was that DBS stimulation in light initiates a level of 2-dim movement similar to the level of 2-dim movement in dark, generally (i.e., in non-stimulated dark). [We have also calculated from the incremental (per sec) changes in angle and the time-evolving winding number, and verified that stimulation initiates spatial movement and not mere spinning in place. (not included in the Results)]

## Discussion

The statistically discoverable principles of the impact of DBS on Generalized Arousal (GA) behavior are not well understood, yet, at the same time, its use in the treatment of a diverse range of diseases is rapidly expanding. The present paper makes an important and vital contribution by identifying, with statistical criteria, the relationship of DBS parameters to induced motor activity (which serves as a correlate for behavior).

In the present work, we have established that DBS does stimulate movement and have quantified the degree of responsiveness to the stimulation parameters (amperage and frequency). Stimulation at 100 *μ*A produced a significant increase in activity above 75 *μ*A (shown to be equivalent to baseline) and 125 *μ*A a significant increase above 100 *μ*A. A key conclusion was that there is a highly significant synergism at the combined 125*μ*A and 125 Hz levels; 125 *μ*A was the highest current, but 125 Hz was mid-level in the (50, 125, 175, 225 Hz) range. This could have a significant impact on the practical use of DBS as a treatment for a variety of diseases. A key concept in the present modeling was the identification of the importance of two-dimensional versus one-dimensional movement. It is 2-D movement, not 1-D, which responds to DBS stimulation. It is 2-D movement, not 1-D, which differs between light and dark. The proportion of 2-D movement in the dark is much greater than the proportion in light. The present experiments revealed that the proportion of 2-D movement initiated by stimulation in light during was similar to that of 2-D movement in non-stimulated dark. These factors, as whole, suggest that stimulation activates in light a manner of movement (2-D movement) that is more commonly, nocturnal. Not only do the above conclusions have important practical consequences for brain arousal, but the methodology developed to draw such inferences, itself, has broad potential.

The methodology is applicable to studies, broadly, for which the data consists of measurements of animal motor activity over time. One cannot apply traditional statistical methods to time-dependent processes, unless the time-varying structure is taken into account. Moreover, as a general statement, if one observes a time-dependent process, for which the time-dependency itself is changing (e.g., circadian rhythm), then, without additional knowledge, very little can be inferred about the underlying structure. If one observes a large number of animals (e.g., 15–20), under identical conditions, one can potentially avoid the time-varying issue. However, if a large number of animals is not observed, then a much different approach is required. One must begin the modeling at the level of the *individual* animal, and build up from there. The key assumption (justified in **Methods**) is that of local stationarity, specifically, *piecewise stationarity*. In such a setting, methods such as shuffling or permutation tests are not valid and bootstrap methods are difficult to implement. If one uses the data in a manner that does not adhere to the time-dependency, one can often end up mistakingly inferring that there is an experimental effect, when in fact what was implicitly being tested (and rejected) was that the data was IID (which it is not).

In the present approach, a method was developed to compare the activity in the stimulation interval to that in neighboring intervals, in a manner consistent with the inherent local stationarity of mouse motor activity, which is highly influenced by light and dark. The developed methods, utilizing piecewise stationarity, allowed one to calculate statistics of motor activity over a segment of time, and, most importantly, to obtain accurate and justified standard errors for those statistics, again for an individual animal. The methods then allowed one to combine across individual animals, reaching the level of desired inference (drawing conclusions based upon the full data). Because the variances at different times of day and/or different DBS parameters, are significantly different (e.g., at times, a factor of 5–10), Analysis of Variance and nonparametric tests are not applicable, but other multiple comparison tests (Dunnett’s C test) are applicable.

The conclusions of the present paper will aid in our understanding of the manner by which the CNS arousal pathways initiate various forms of behavior. In addition, the methodology developed for this work provides the experimentalist with justified methods for testing hypotheses in the common neuroscience framework in which animal motor activity is measured.

## References

[1] Kahan J, Mancini L, Urner M, Friston K, Hariz M, Holl E et al (2012). Therapeutic subthalamic nucleus deep brain stimulation reverses cortico-thalamic coupling during voluntary movements in Parkinson’s disease. PLoS One;7(12):e50270 10.1371/journal.pone.0050270 23300524 PMC3530565

[2] Wichmann T, DeLong MR. (2006). Deep brain stimulation for neurologic and neuropsychiatric disorders. Neuron; 52:197–204. 10.1016/j.neuron.2006.09.022 17015236

[3] Smith AC, Shah SA, Hudson AE, Purpura KP, Victor JD, Brown EN, Schiff ND. (2009). A Bayesian statistical analysis of behavioral facilitation associated with deep brain stimulation. Journal of Neuroscience Methods; 183:267–76. 10.1016/j.jneumeth.2009.06.028 19576932 PMC2743761

[4] Schiff ND, Giacino JT, Kalmar K, Victor JD, Baker K, Gerber M, Fritz B, Eisenberg B, Biondi T, O’Connor J, Kobylarz EJ, Farris S, Machado A, McCagg C, Plum F, Fins JJ, Rezai AR. (2007). Behavioural improvements with thalamic stimulation after severe traumatic brain injury. Nature; 8 2;448(7153):600–3. [Erratum in: *Nature*; 2008 Mar 6;452(7183):120.] 10.1038/nature06041 17671503

[5] Schiff ND. (2009). Central thalamic deep-brain stimulation in the severely injured brain: rationale and proposed mechanisms of action. Ann N Y Acad Sci; 1157:101–16 10.1111/j.1749-6632.2008.04123.x 19351360

[6] Pfaff DW. (2006). Brain Arousal and Information Theory. Harvard University Press Cambridge.

[7] Leshner A. and Pfaff DW. (2011). Quantification of behavior. Proc Natl Acad Sci USA; 108(Suppl. 3):15537–41. 10.1073/pnas.1010653108 21914850 PMC3176606

[8] Benjamini Y., Fonio E., Galili T., Havkin GZ. and Golani I. (2011) Quantifying the buildup in extent and complexity of free exploration in mice. Proc Natl Acad Sci USA; 108 (Suppl. 3):15580–15587. 10.1073/pnas.1014837108 21383149 PMC3176604

[9] Quinkert AW, Schiff ND, Pfaff DW. (2010). Temporal patterning of pulses during deep brain stimulation affects central nervous system arousal. Behav Brain Res; 214(2):377–85. 10.1016/j.bbr.2010.06.009 20558210

[10] Quinkert AW, Vimal V, Weil ZM, Reeke GN, Schiff ND, Banavar JR, Pfaff DW. (2011). Quantitative descriptions of generalized arousal, an elementary function of the vertebrate brain. Proc Natl Acad Sci USA; 3:15617–23. 10.1073/pnas.1101894108 PMC3176607 21555568

[11] Quinkert AW, Pfaff DW. (2012). Temporal patterns of deep brain stimulation generated with a true random number generator and the logistic equation: effects on CNS arousal in mice. Behav Brain Res; 229(2):349–58. 10.1016/j.bbr.2012.01.025 22285420 PMC3721317

[12] Ombao H., Raz J, von Sachs R. and Mallow B. (2001). Automatic statistical analysis of bivariate non-stationary time series. J Amer Statist Assoc; 96; 543–560. 10.1198/016214501753168244

[13] Huang H., Ombao H. and Stoffer D. (2004). Classification and discrimination of non-stationary time series using the SLEX model. J Amer Statist Assoc; 99; 763–774. 10.1198/016214504000001105

[14] Diggle PJ. and Fisher NI. (1991). Nonparametric comparisons of cumulative periodograms. J R Statist Soc, Ser C; 40; 423–434.

[15] Priestley MB. and Subba Rao T. (1969). A test of non-stationarity of time-series. J R Statist Soc, Ser B; 31; 140–149.

[16] Billingsley P. (1968). Convergence of Probability Measures. Wiley, New York.

[17] Benjamini Y. and Hochberg Y. (1995). Controlling the False Discovery Rate: A Practical and Powerful Approach to Multiple Testing. J R Statist Soc, Ser B; 57(1):289–300.

[18] Benjamini Y and Yekutieli D. (2001). The control of the false discovery rate in multiple testing under dependency. Annals of Statistics; 29(4):1165–88.

[19] Shumway RH and Stoffer DS. (2011). Time Series Analysis and Its Applications: With R Examples. 3rd Edition Springer, New York.

[20] Grenander U and Rosenblatt M. (1957). Statistical Analysis of Stationary Time Series. Wiley, New York.

[21] Priestley MB. (1981). Spectral Analysis and Time Series. Academic Press, New York.

[22] Kunsch HR. (1989). The jackknife and the bootstrap for general stationary observations, Annals of Statistics; 17; 1217–1261. 10.1214/aos/1176347265

[23] Lahiri SN. (2003). Resampling Methods for Dependent Data. Springer-Verlag, New York.

[24] Scheffe H. (1959). The Analysis of Variance. John Wiley and Sons, New York.

[25] Dunnett C. (1980). Pairwise multiple comparisons in the unequal variance case. J Amer Statist Assoc; 75; 373, 796–800. 10.1080/01621459.1980.10477551

